# Coordination of plant mitochondrial biogenesis: keeping pace with cellular requirements

**DOI:** 10.3389/fpls.2013.00551

**Published:** 2014-01-08

**Authors:** Elina Welchen, Lucila García, Natanael Mansilla, Daniel H. Gonzalez

**Affiliations:** ^1^Instituto de Agrobiotecnología del Litoral–Consejo Nacional de Investigaciones Científicas y Técnicas-Universidad Nacional del LitoralSanta Fe, Argentina; ^2^Cátedra de Biología Celular y Molecular, Facultad de Bioquímica y Ciencias Biológicas, Universidad Nacional del LitoralSanta Fe, Argentina

**Keywords:** mitochondrial dynamics, mitochondrial biogenesis, retrograde signal, post-transcriptional gene regulation, site II, coordination

## Abstract

Plant mitochondria are complex organelles that carry out numerous metabolic processes related with the generation of energy for cellular functions and the synthesis and degradation of several compounds. Mitochondria are semiautonomous and dynamic organelles changing in shape, number, and composition depending on tissue or developmental stage. The biogenesis of functional mitochondria requires the coordination of genes present both in the nucleus and the organelle. In addition, due to their central role, all processes held inside mitochondria must be finely coordinated with those in other organelles according to cellular demands. Coordination is achieved by transcriptional control of nuclear genes encoding mitochondrial proteins by specific transcription factors that recognize conserved elements in their promoter regions. In turn, the expression of most of these transcription factors is linked to developmental and environmental cues, according to the availability of nutrients, light–dark cycles, and warning signals generated in response to stress conditions. Among the signals impacting in the expression of nuclear genes, retrograde signals that originate inside mitochondria help to adjust mitochondrial biogenesis to organelle demands. Adding more complexity, several nuclear encoded proteins are dual localized to mitochondria and either chloroplasts or the nucleus. Dual targeting might establish a crosstalk between the nucleus and cell organelles to ensure a fine coordination of cellular activities. In this article, we discuss how the different levels of coordination of mitochondrial biogenesis interconnect to optimize the function of the organelle according to both internal and external demands.

## MITOCHONDRIA AS DYNAMIC ORGANELLES

**FIGURE 1:**
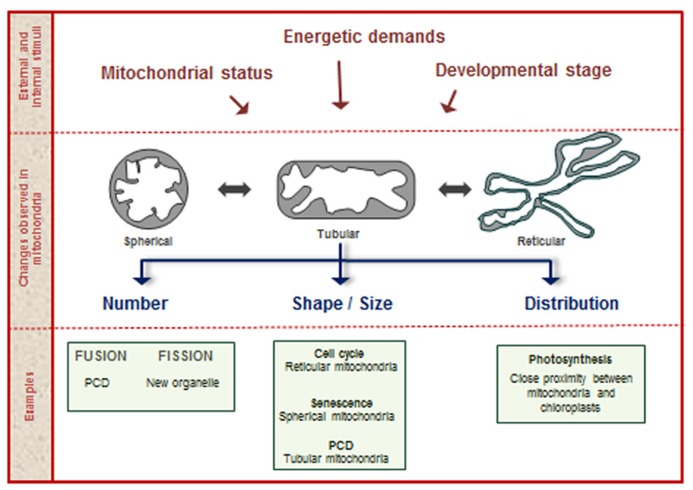
**Mitochondrial dynamics.** During the plant life cycle, mitochondria change in shape, size, and number, according to numerous stimuli provided by internal and external factors. Examples of processes related with changes in mitochondrial structure are shown in the lower part of the figure.

New mitochondria arise from the fission of preexisting organelles. Mitochondria can also undergo fusion events, where two or more organelles converge into a single new one. Mitochondria are among the most plastic organelles of cells, alternating their architecture and distribution throughout the cytosol in order to carry out cellular functions. Moreover, mitochondrial number and morphology vary among different organisms and depending on the physiological and developmental conditions of the cell. Plant cells typically contain several hundred physically discrete mitochondria. For example, *Arabidopsis* mesophyll cells contain 200–300 discrete mitochondria, while tobacco mesophyll protoplasts contain 500–600 (Logan, 2010; Preuten et al., 2010). The processes related with changes in mitochondrial shape, size, and number are known as mitochondrial dynamics (Scott and Logan, 2010; **Figure 1**). This is a regulated process in plants, essential for the exchange of metabolic, genetic, and protein contents and to modulate mitochondrial bioenergetics, ATP production, autophagy, plant cell death (PCD), and connections with the cell cycle (Hyde et al., 2010; Westermann, 2012; Schwarzländer et al., 2012).

Besides the tight regulation required during changes associated with mitochondrial dynamics, it is evident that these changes imply coordinated responses of the many genes that encode organelle components. As proposed in mammals, the regulation of mitochondrial dynamics occurs at two interconnected levels. One of the coordination events is known as “organellar” control and is represented by the control that the mitochondrion exerts on itself. This local process, mainly represented by post-translational protein modifications, alters the microenvironment of the individual mitochondrion modifying its ability to fuse, divide, or move through the cell (Hyde et al., 2010). The “global” control is enforced by the cellular environment, when nuclear-encoded proteins, ions, and second messengers modify mitochondrial protein composition, thus remodeling mitochondrial populations. Changes in mitochondrial dynamics during the cell cycle are the most common examples of global control (Hyde et al., 2010).

The main goal of mitochondrial dynamics would be to optimize mitochondrial function according to the specific energetic needs of the cell. As mentioned, one of the first evidences relating mitochondrial dynamics and function comes from the observation of plant mitochondria at different stages of the cell cycle. The observation of electron micrographs of serial thin-sections prepared from *Arabidopsis* apical meristems at various developmental stages demonstrated the presence of large sheet-like mitochondria that undergo characteristic morphological and architectural changes during the cell cycle (Seguí-Simarro and Staehelin, 2009). The authors proposed that large, reticulate mitochondria provide an efficient means to deliver ATP for cell cycle and cytokinesis and enable efficient mixing and recombination of mitochondrial DNA (mtDNA; Seguí-Simarro et al., 2008). In this sense, it has been postulated that the plant chondriome is organized as a discontinuous whole and that there is a requirement for movement of the organelles, predominantly trough the actin cytoskeleton, to drive the meeting of discrete mitochondria in order to allow the exchange of mtDNA (Logan, 2006, 2010).

Another evidence linking dynamics to function was obtained in plant photosynthetic tissues through the observation of the co-localization or close proximity between mitochondria and chloroplasts (Logan and Leaver, 2000). More recently, Islam and Takagi (2010) showed the existence of changes in mitochondrial cellular location associated with chloroplast movements under different light regimes. In the dark, mitochondria were distributed randomly in palisade mesophyll cells. However, under different light intensities mitochondria moved coordinately with chloroplasts, suggesting that they either follow a specific signal or become physically associated with chloroplasts through the cytoskeleton. Although there is no specific evidence on this, it is assumed that this close association facilitates the exchange of gases and metabolites required for the maintenance of efficient photosynthesis (Islam and Takagi, 2010).

In addition, physical interactions between mitochondria and the endoplasmic reticulum (ER) were observed in mammalian cells. These contacts are established through proteins exposed in the surface of the organelles and allow the exchange of lipids and calcium (Kornmann, 2013). They also mark the sites where mitochondrial division will take place, thus suggesting a role of the ER in mitochondrial dynamics (Friedman et al., 2011).

Mitochondrial dynamics and morphology also change during different growth phases of cells in culture. Healthy, growing cells are characterized by a typical reticular arrangement of mitochondria. When cells enter senescence, the network disintegrates into very small mitochondria. Finally, giant mitochondria are observed when high levels of cell death are reached in the culture (Zottini et al., 2006). Other authors also demonstrated that one of the first evidences of the onset of PCD induced by reactive oxygen species (ROS) accumulation is the morphological change from tubular to spherical mitochondria in *Arabidopsis* (Yoshinaga et al., 2005). All these data reinforce the idea that mitochondria are dynamic organelles, changing their number, form, and position inside the cell according to developmental stage, type of tissue, cell cycle phase, energetic cell demand, external stimuli, and PCD. This dynamic behavior is likely a consequence of their central role in many cellular activities.

## MITOCHONDRIAL BIOGENESIS

Usually, mitochondrial biogenesis is defined as the process by which “new” mitochondria are formed in the cell. Since “new” mitochondria arise from pre-existing ones, the term mitochondrial biogenesis refers rather to the processes involved in the synthesis and assembly of new mitochondrial components. As a consequence, mitochondrial biogenesis is closely associated with mitochondrial dynamics, since many of the processes referred in the previous section most likely imply changes in mitochondrial composition and/or number. The synthesis and assembly of mitochondrial components is a complex process that requires the expression of two different genomes, the synthesis of proteins in two different compartments, the transport of proteins and/or their insertion into membranes and, frequently, the assembly of proteins and cofactors into multimeric complexes.

The complex nature of this process and of the functions performed by mitochondrial components suggests the idea that mitochondrial biogenesis must highly coordinated. In the following sections, we will try to summarize the knowledge available about the factors and mechanisms involved in the coordination of mitochondrial biogenesis in plants. The term coordination is used here in two different ways. One is to imply that the different components of the organelle must be coordinately synthesized to assemble “new” mitochondria. The other one refers to the fact that mitochondrial biogenesis must be coordinated with other cellular activities, considering the central role of these organelles in eukaryotic cells. While gene expression within mitochondria is essential for mitochondrial biogenesis, we will not refer to the expression mechanisms of mitochondrial genes, which have been extensively reviewed (Binder and Brennicke, 2003; Gagliardi and Binder, 2007; Liere et al., 2011). We will rather focus on the factors, either from outside or inside the organelle, that impact on the expression of nuclear genes encoding mitochondrial proteins and try to understand the mechanisms involved in coordinating gene expression and mitochondrial biogenesis both among different mitochondrial components and with the rest of the cell and the environment.

### FROM OUTSIDE TO INSIDE: REGULATION OF MITOCHONDRIAL BIOGENESIS BY EXTERNAL FACTORS

#### Factors from outside the organelle that influence mitochondrial biogenesis

Mitochondria are not static and are constantly changing in form, volume, number, and composition inside cells (Logan, 2010; Preuten et al., 2010; Peters et al., 2012; Lee et al., 2013). These changes are not random, but rather respond to different factors, like plant organ, tissue and developmental stage, environmental stimuli, and cell energy and metabolic demands, among others (Howell et al., 2009; Law et al., 2012). In agreement with the central role of mitochondria in cellular metabolism and as energy suppliers during developmental processes and cellular differentiation, their biogenesis is highly regulated at different stages of development, and according to organ and tissue type. As an example of coordination, similar patterns of transcript accumulation for the nuclear encoded cytochrome *c* gene and genes encoded in the mitochondrial genome were reported during flower development in sunflower (Ribichich et al., 2001). This is in agreement with a wealth of information indicating that most components of the oxidative phosphorylation machinery show higher expression in flowers, particularly in anthers (Welchen et al., 2004; Gonzalez et al., 2007; Peters et al., 2012), maybe in relation with increased energy demand during pollen maturation. In addition, it has been shown that the relative abundance of the respiratory complexes varies according to tissue type. In this sense, Peters et al. (2012) showed that the ratio of protein components of Complexes I and II differ in photosynthetic tissues with respect to non-green organs such as roots and calli. Other authors found differences in respiratory rates with different substrates and in activities of specific enzymes in mitochondria isolated from non-photosynthetic cell cultures or from photosynthetic shoots (Lee et al., 2008, 2011).

Another process in which mitochondria play a crucial role is during germination. Studies performed in rice demonstrated that respiration increases rapidly during the first 24 h after imbibition. This is due to the execution of a program where the components of the protein import machinery are already present in promitochondrial structures to facilitate a rapid rate of mitochondrial biogenesis after imbibition (Howell et al., 2006; Taylor et al., 2010). In *Arabidopsis*, Law et al. (2012) made an exhaustive analysis to elucidate how mitochondrial biogenesis takes place during germination and early seedling growth. They showed the existence of changes in mitochondrial number, size, and morphology after 12 h of imbibition in continuous light, prompted by an increase of mitochondrial protein expression, and established a model for mitochondrial biogenesis during germination in which an early increase in the abundance of transcripts encoding proteins involved in transcription, translation and replication precedes a later cascade of expression of genes encoding bioenergetic and metabolic functions (Law et al., 2012). The examples mentioned above indicate that factors related with plant developmental or cell differentiation programs influence mitochondrial biogenesis either globally or specifically, most likely to adjust mitochondrial functions to cellular demands.

The expression of nuclear respiratory genes is also regulated by numerous external factors not directly related with plant developmental programs, such as nutrient availability (Welchen et al., 2002, 2004; Giegé et al., 2005), hormones (Comelli et al., 2009), light/dark conditions (Welchen et al., 2002), the diurnal cycle (Gibala et al., 2009; Lee et al., 2010), growth conditions generating oxidative stress (Ho et al., 2008; Obata et al., 2011; Tan et al., 2012; Van Aken and Whelan, 2012; Giraud et al., 2012; Lee et al., 2013), and abiotic or biotic stress conditions (Attallah et al., 2007; Livaja et al., 2008; Cvetkovska and Vanlerberghe, 2013). Due to the multiple interconnections of mitochondria with other organelles (peroxisomes, chloroplasts, ER, and the nucleus), it is difficult to identify if these external signals impact directly in mitochondrial biogenesis or act through changes in the cellular environment.

#### Molecular mechanisms of nuclear gene expression involved in mitochondrial biogenesis

**FIGURE 2:**
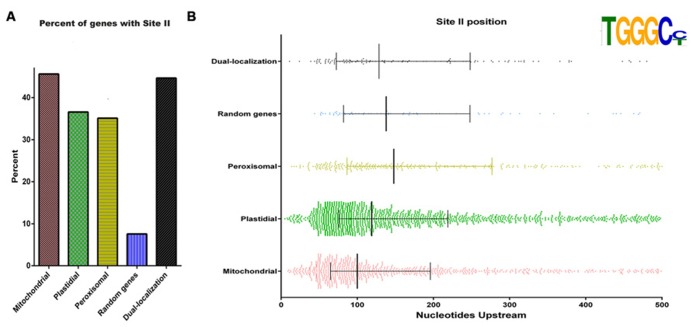
**Site II elements are enriched in genes encoding mitochondrial proteins and are present in many nuclear genes encoding plastid and peroxisomal components. (A)** Proportion of genes with site II elements among *Arabidopsis* nuclear genes encoding proteins destined to different cell compartments. Gene sets were taken from Law et al. (2012). Dual targeted proteins were taken from Xu et al. (2013b). The term “Random genes” refers to a set of 490 genes picked at random. **(B)** Location of site II elements respective to the transcription start site in different sets of genes. Vertical lines represent the first, second, and third quartiles, respectively.

Mitochondrial respiratory activity is carried out by a series of multimeric complexes consisting of subunits encoded either in the nucleus or in the organelle. Based on this, the assembly of these complexes is a finely regulated process that requires the coordination of the expression of multiple genes (Giegé et al., 2005; Gonzalez et al., 2007). Giegé et al. (2005), using *Arabidopsis* cells grown under starvation conditions, proposed a model suggesting that mitochondrial encoded proteins are always in excesses inside the organelle, while proteins encoded in the nucleus are the limiting factors. In this sense, it was shown that, for nuclear genes, coordination takes place at the transcriptional level, most likely through the interaction of transcription factors with common regulatory elements present in the respective promoters (Welchen and Gonzalez, 2006; Welchen et al., 2009; Comelli et al., 2009; Comelli and Gonzalez, 2009). Coordinated expression of most nuclear genes encoding components of the different respiratory complexes was observed as a consequence of changes in carbohydrate supply or metabolism, photo-destruction of chloroplasts, inhibition of cellulose synthesis, and release from dormancy and germination (Gonzalez et al., 2007). Particularly, carbohydrate levels would operate to balance the biogenesis of the photosynthetic apparatus and the respiratory chain, which are involved in their synthesis and use, respectively. It has been demonstrated that the expression of a majority of nuclear genes for mitochondrial proteins is controlled by elements known as site II, which are either responsible for basal gene expression (Welchen and Gonzalez, 2006; Attallah et al., 2007) or modify the magnitude of the response under different growth conditions (Welchen et al., 2009; Mufarrege et al., 2009; Comelli et al., 2009, 2012). Indeed, over 80% of genes encoding constituents of Complexes I, III, IV, and V of the mitochondrial respiratory machinery in *Arabidopsis* and rice have site II elements in their promoter regions (Welchen and Gonzalez, 2006). It was postulated that site II elements are recognized by transcription factors from the TCP family (Trémousaygue et al., 2003; Martín-Trillo and Cubas, 2010; Uberti Manassero et al., 2013), which modulate processes related with cell proliferation and growth. This would provide a link for integrating gene expression with cellular demands (Gonzalez et al., 2007). Interestingly, site II elements are also enriched in genes encoding ribosomal proteins and are present in many nuclear genes encoding plastid and peroxisomal components (**Figure 2A**). This suggests that site II elements may be involved in the biogenesis of several cell components under conditions of active cell or tissue growth. This global mode of regulation may superimpose with more specific factors to modulate responses of subsets of genes. It is interesting that site II elements seem to accumulate closer to the transcription start site in genes encoding mitochondrial proteins than in those encoding components of other organelles (**Figure 2B**). In addition, other enriched elements, like those identified by Leister et al. (2011), may help to impose specificity to the response.

Lee et al. (2010) also showed that most genes that exhibit a strong response to changes in light/dark cycles have site II elements in their promoter regions. In contrast, genes without site II elements did not show clear cycling transcript abundance patterns. Giraud et al. (2010) showed that interactions between site II elements and TCP proteins are able to coordinate the expression of nuclear-encoded genes for mitochondrial proteins with the circadian clock. Recently, it was shown that one member of the TCP family in *Arabidopsis*, AtTCP8, is able to interact in the nucleus with the promoter region of *PNM1*, a gene that encodes a pentatricopeptide repeat (PPR) protein that localizes to the nucleus and mitochondria (Hammani et al., 2011a). Since PPR proteins participate in the expression of plant mitochondrial genes, it has been postulated that the AtTCP8-PNM1 interaction may operate to adjust the expression of both genomes during mitochondrial biogenesis (Hammani et al., 2011b).

Among other transcription factors involved in the expression of nuclear genes encoding mitochondrial components, we can mention transcription factors from the bZip, AP2/ERF, bHLH, and trihelix families. Specifically, the incorporation of the abscisic acid (ABA)-responsive element binding factor AREB2/ABF4 into complexes involved in the expression of the *COX5b-1* and *Cytc-2* genes may have allowed the diversification of regulatory mechanisms (Comelli et al., 2009, 2012; Comelli and Gonzalez, 2009; Welchen et al., 2009). In an extreme case of diversification, a gene encoding an isoform of a succinate dehydrogenase (Complex II) subunit that is expressed specifically in seeds has been shown to be regulated by three B3 domain transcription factors involved in seed maturation (Elorza et al., 2004; Roschzttardtz et al., 2009).

As mentioned above, transcriptional regulation is a main regulatory point in the expression of nuclear encoded genes associated with respiration. However, there are several examples in which protein levels do not perfectly correlate with relative transcript abundance. One example of post-transcriptional regulation occurs during germination of *Arabidopsis* seeds and in responses to oxygen availability, where levels of import machinery components change markedly, while their transcript levels remain relatively stable (Howell et al., 2006, 2007; Law et al., 2012; Murcha et al., 2013). Interestingly, transcript abundance of the components of the electron transport chain display a high level of similarity with protein abundance, in marked contrast with the observation made for mitochondrial import components (Law et al., 2012). This example shows that the expression of different sets of proteins encoded by nuclear genes may be regulated at different levels even in response to the same process. In this case, it is logical to assume that post-transcriptional regulation of the import machinery reassures a rapid increase of its components that are required for the incorporation of other nuclear encoded proteins during mitochondrial biogenesis.

Even if initial studies suggested that mitochondrial-encoded subunits are present in excess and nuclear-encoded proteins are the limiting factors (Giegé et al., 2005), a recent study by Kwasniak et al. (2013) demonstrated that the synthesis of mitochondrial proteins may become a limiting factor under certain situations. These authors showed that mutations in the nuclear *RPS10* gene produce an increase in mitoribosomes but a decrease in the abundance of all oxidative phosphorylation complexes. This is due to an increased synthesis of mitochondrial encoded ribosomal proteins, while the opposite occurs for mitochondrial encoded respiratory chain subunits. Although these observations were made with a mutant plant, it can be envisaged that certain conditions may impose limitations to mitochondrial translation and this may impact in the differential synthesis of mitochondrial components. The findings highlight the importance of different levels of regulation in establishing the synthesis of mitochondrial protein complexes.

In animals, a recently proposed mechanism for the regulation of mitochondrial biogenesis involves the participation of microRNAs (miRNAs; Li et al., 2012; Yamamoto et al., 2012). These are a class of 20- to 24-nucleotide endogenous small non-coding RNAs that can bind to mRNAs, thereby inhibiting mRNA translation or promoting RNA degradation. This miRNA-mediated regulation is sequence-specific and occurs at the post-transcriptional level (Li et al., 2012). In plants, miRNAs control the expression of genes involved in many different processes (Rogers and Chen, 2013). Several animal miRNAs modulate the expression of mitochondrial proteins encoded by nuclear genes (Li et al., 2012; Tomasetti et al., 2013) and miRNAs have also been found in mitochondria, then receiving the name of “mitomiRs” (Bian et al., 2010). An example showing how mitomiRs may be involved in regulating mitochondrial functions comes from studies with miR181c. It has been reported that this miRNA encoded in the nucleus can enter mitochondria and target the genome, causing remodeling of Complex IV and mitochondrial dysfunction (Das et al., 2012).

In plants, there is little evidence on the involvement of miRNAs in mitochondrial biogenesis. By using a computational approach, Kamarajan et al. (2012) identified seven potential mitochondrial miRNA targets, but functional studies on the effect of these miRNAs were not provided. Regarding the expression of nuclear genes encoding mitochondrial proteins, an example of the involvement of miRNAs in regulation is provided by miR398, which binds to the 5′-UTR of the *Cox5b-1* transcript (Jones-Rhoades and Bartel, 2004). This miRNA is involved in regulating the expression of genes encoding copper binding proteins located in several cell compartments according to copper availability and may thus act to coordinate the biogenesis of Complex IV with other cellular processes. However, a clear demonstration that miR398 levels affect Complex IV assembly has not been provided.

## FROM OUTSIDE TO MORE THAN ONE SITE: DUAL TARGETING OF PROTEINS

The term “dual targeting” refers to the phenomenon where a single protein can be distributed and/or localized in more than one subcellular compartment. This event may be a solution found through evolution to increase the number of cellular functions without increasing the number of genes. It can also be thought as a dynamic process that enables cells to adapt and coordinate their functions in a flexible way according to cellular and environmental demands (Yogev and Pines, 2011). The fact that sometimes both dual targeted and specific protein isoforms exist in the same organelle suggests that, rather than for economy, the cell may use in some cases this mechanism for regulatory purposes. Dual targeting can be achieved via two basic mechanisms: alternative transcription initiation or splicing and ambiguous targeting signals recognized by the organelle receptors and import machinery (Peeters and Small, 2001; Yogev and Pines, 2011).

Contrary to other eukaryotes, very few instances of nucleo-mitochondrial proteins have been described in plants. However, because the primary function of the mitochondrion is the respiratory activity performed by a series of multisubunit complexes whose expression must be coordinated between the nucleus and mitochondria, it seems obvious that the dual localization of proteins in both compartments may have an important role. A well described example is represented by PNM1, an *Arabidopsis* PPR protein identified in both mitochondria and the nucleus (Hammani et al., 2011a). PNM1 may be related to organellar translation processes because it is associated with mitochondrial polysomes (Hammani et al., 2011a). In addition, it interacts in the nucleus with the transcription factor TCP8 that belongs to the TCP family (Hammani et al., 2011b) whose members may participate in the expression of nuclear encoded mitochondrial proteins (Welchen and Gonzalez, 2006; Gonzalez et al., 2007). In the absence of PNM1, nuclear genes encoding mitochondrial proteins show increased transcript levels. This may suggest that PNM1 acts to coordinate the expression of genes for mitochondrial components located in both compartments (Hammani et al., 2011b; Duchêne and Giegé, 2012).

While information about proteins present in mitochondria and the nucleus is scarce, more than 100 proteins were shown to be targeted to mitochondria and plastids (Carrie et al., 2009; Carrie and Small, 2013; Xu et al., 2013a). The number of proteins actually targeted to both organelles may be considerably higher than this, since a recent report has shown that even many proteins predicted to be targeted to a single organelle are imported into both *in vitro* and *in vivo* (Baudisch et al., 2014). Although it has not been clearly demonstrated, it can be speculated that the presence of proteins which are able to fulfill related functions in both organelles is a strategy acquired through evolution to coordinate processes related with energy homeostasis in the cell. There are also examples of proteins targeted to both mitochondria and peroxisomes, like type II NAD(P)H dehydrogenases and Mia40 (Xu et al., 2013b). Mia40 is a mitochondrial protein involved in the transport of CX_9_C and CX_3_C proteins in other organisms. Inactivation of *Arabidopsis* Mia40 produces changes in mitochondrial and peroxisomal proteins (Carrie et al., 2010), but there does not appear to be any effect on the assembly of Complex IV, that requires several CX_9_C and CX_3_C proteins for its biogenesis, suggesting that either a different import system is used by these proteins in plants or another protein fills the Mia40 function.

## FROM INSIDE TO OUTSIDE: IMPACT OF MITOCHONDRIAL HOMEOSTASIS ON MITOCHONDRIAL BIOGENESIS AND NUCLEAR GENE EXPRESSION

### FUNCTIONAL INTERCONNECTIONS BETWEEN THE MITOCHONDRIAL IMPORT MACHINERY AND THE RESPIRATORY CHAIN MODULATE MITOCHONDRIAL BIOGENESIS

An important step in mitochondrial biogenesis is the import of proteins encoded in the nucleus into the organelle. In addition to respiratory complexes, the mitochondrial inner membrane (IM) harbors the protein translocases required for the import of mitochondrial precursor proteins. The import of respiratory chain precursor proteins across and into the IM requires the membrane potential generated by the proton pumping complexes of the respiratory chain. In addition, certain components of the import machinery are physically linked to respiratory chain complexes. This physical interaction between components of both systems most likely produces also a functional connection. It can be envisaged that these interactions provide a mechanism for the coordination of mitochondrial biogenesis and function.

Among the examples of the importance of physical interconnections between the import machinery and the respiratory chain in plants, one of the best characterized is that of the α and β subunits of the mitochondrial processing peptidase (MPP), which are components of the cytochrome *b*c_1_ complex (Complex III; Brumme et al., 1998). The second type of physical interaction between the import machinery and the respiratory chain is represented by the multiple interactions observed between the TIM17:23 complex and Complexes I, III, and IV in *Arabidopsis* and yeast. In yeast, the TIM17:23 complex has been shown to form dynamic supercomplexes with both the TOM40 channel and Complexes III and IV via Tim21. This interconnection would guarantee an efficient protein translocation process at contact sites by utilizing the membrane potential of the IM (Stuart, 2008; Duncan et al., 2013).

In *Arabidopsis*, Tim23-2 was shown to associate with Complex I. Independent Complex I knock-out lines show increased abundance of Tim23-2. In turn, Tim23-2 overexpressing lines show a decrease in Complex I abundance and a two to threefold increase in transcript levels of mitochondrial genes, organelle translation, and protein import. Nuclear genes encoding mitochondrial proteins also display increased expression, in particular genes encoding proteins involved in mitochondrial biogenesis (Wang et al., 2012; Murcha et al., 2012). In addition, overexpression of SD3, a protein with high similarity to yeast Tim21 which is part of the TIM23 complex, increases cotyledon size, ATP levels, and the expression of genes for several subunits of respiratory chain Complexes III and IV. This constitutes another example of the role of the import machinery in the control of respiratory activity and also suggests the existence of a link between mitochondrial activity and the control of organ size through cell growth and proliferation (Hamasaki et al., 2012).

### MITOCHONDRIAL RETROGRADE SIGNALING: MITOCHONDRIAL STATUS MODULATES NUCLEAR GENE RESPONSES

Respiratory chain biogenesis implies the coordination between mitochondria and the nucleus since the different components are encoded either in the nuclear or the mitochondrial genome. In this coordinated network, mitochondria need to generate signals to modify nuclear gene expression according to organelle and cellular requirements. Signaling pathways from organelles to the nucleus are referred to as Retrograde Signaling pathways. While several relevant players in chloroplast retrograde signaling have been extensively characterized (for reviews see Leister et al., 2011; Kleine and Leister, 2013), relatively little is known about mitochondrial retrograde signaling (MRS). Even if a wealth of information published in the last 10 years has clearly shown that mitochondrial perturbations have a consequence in nuclear gene expression (Clifton et al., 2005; Giraud et al., 2009; Meyer et al., 2009; Shedge et al., 2010; Busi et al., 2011; Schwarzländer et al., 2012), the signals that operate these changes remain unclear. Considering that chloroplasts and mitochondria are closely connected, signaling pathways involved in chloroplast retrograde regulation need to be explored in the context of mitochondria to get new insights into MRS.

How mitochondria produce and transmit the signal is a critical step to clarify MRS pathways. Schwarzländer and Finkemeier (2013) extensively revised putative mitochondrial signals and focused in redox and bioenergetic processes housed in the mitochondrion as strategic sources of signals to transmit mitochondrial state. In line with this, metabolites like ascorbate and glutathione have been postulated as players in chloroplast retrograde signaling (Foyer and Noctor, 2011). These metabolites are considered as potential molecular signals for retrograde signaling due to their capacity to transfer redox information. In addition, an intermediate metabolite in the tetrapyrrole pathway (Mg-protoporphyrin IX) has been shown to be a signaling molecule from the chloroplast (Mochizuki et al., 2008). More recently, citrate has been proposed as a signaling molecule that affects nuclear gene expression by unknown mechanisms (Finkemeier et al., 2013). Finding response elements in promoters of nuclear-encoded mitochondrial genes and, particularly, the discovery of specific marker genes for mitochondrial dysfunction are the first steps to get more insight into MRS in plants. In the next short sections we briefly revise the latest advances in MRS, considering two steps of this intricate process: downstream (nuclear gene expression) and upstream (signals triggered by mitochondria) components.

#### Downstream components: transcription factors involved in MRS

*AOX1a*, a gene encoding an isoform of the alternative oxidase, is a classic example of a gene regulated by mitochondrial signals. Chemical inhibition of electron transport through the respiratory chain induces the expression of AOX1a (Zarkovic et al., 2005; Vanlerberghe, 2013). Analysis of proteins that interact with the *AOX1a* promoter showed that the transcription factor ABA INSENSITIVE 4 (ABI4) may be an important player in regulating the expression of AOX1a (Clifton et al., 2005; Giraud et al., 2009). ABI4 acts as a repressor, since *AOX1a* expression is induced in *ABI4* mutant plants (Giraud et al., 2009). The fact that the respiratory chain inhibitor rotenone induces *AOX1a* expression in wild-type but not in *ABI4* mutant plants suggests that ABI4 mediates mitochondrial retrograde signals involved in *AOX1a* expression. This observation also provides a molecular link between mitochondrial and chloroplast retrograde signaling, since ABI4 was also shown to participate in at least two chloroplast retrograde signaling pathways (Koussevitzky et al., 2007). It was also shown that *AOX1a* shares six *cis*-acting regulatory elements that play a role in responses to rotenone and H_2_O_2_ with the external NAD(P)H-dehydrogenase *NDB2* gene (Ho et al., 2008).

A recent transcriptomic analysis considering 27 different mitochondrial and chloroplast perturbations identified a set of 12 nuclear-encoded mitochondrial genes that exclusively respond to mitochondrial perturbations (Van Aken and Whelan, 2012). Among them, the authors identified a new MRS marker, AtMSM1, which has a CHCH domain and resembles Mic17p, a yeast protein located in the mitochondrial intermembrane space. The characteristics of this protein and its putative location suggest that it may not only be a target, but also a transducer of MRS. Several *Arabidopsis* proteins that contain redox active cysteines related in structure to AtMSM1, like AtCOX17 and AtCOX19 among others, are present in the intermembrane space and are induced under several stress conditions (Attallah et al., 2007). This points to the intermembrane space as a site of sensing or transduction of signals that connect mitochondrial state with the rest of the cell.

WRKY transcription factors are involved in several biotic and abiotic stress responses. These factors recognize a responsive element known as the W box. The fact that W boxes are overrepresented in promoters of genes that specifically respond to mitochondrial dysfunction suggests that these factors may participate in MRS (Van Aken and Whelan, 2012). For example, three W-box motifs were identified in the *AOX1a* promoter (Dojcinovic et al., 2005). In addition, genes encoding mitochondrial proteins were overrepresented among genes repressed in plants overexpressing WRKY15, which was suggested as a player in MRS under salt stress, influencing mitochondrial responses regulated by calcium fluxes (Vanderauwera et al., 2012). Since WRKY transcription factors participate in multiple signaling pathways related to stress, the question remains if WRKY15 or any other member of the family is specific for MRS or has a more general role in stress responses. More recently, two other members of the family, WRKY40 and WRKY63, were identified as mediators of retrograde responses related with both chloroplast and mitochondrial perturbations (Van Aken et al., 2013).

Other players in MRS that were recently identified belong to the NAC transcription factor family. ANAC013 and ANAC017 are functionally associated with MRS and bind to a newly identified responsive element (named MDM) present in a set of MRS genes, like *AOX1a* (De Clercq et al., 2013; Ng et al., 2013). Interestingly, these factors are bound to the ER and, at least for ANAC017, its cleavage and migration to the nucleus seems to be related with induction of the *AOX1a* gene by MRS (Ng et al., 2013).

#### Upstream signaling: looking for the molecules that connect mitochondria with nuclear responses

Respiration is essentially a redox process. As a consequence, redox changes are likely involved in signaling mechanisms related with mitochondrial function (Finkemeier et al., 2005; Hicks et al., 2007; Collins et al., 2012). Inhibition or overreduction of the electron transport chain generates an increase in ROS production in mitochondria, mainly at the level of Complexes I and III (Turrenes, 2003; Dröse and Brandt, 2012). ROS are known as signaling molecules in many different cellular processes and may well also function in MRS (Møller and Sweetlove, 2010; Schwarzländer et al., 2012). Different H_2_O_2_-sensitive transcription factors were discovered in plants (Lindermayr et al., 2010; Shaikhali et al., 2008; Shaikhali and Baier, 2010; Viola et al., 2013). Particularly, TCP15, AtbZIP16 and Rap2.4a are involved in the control of nuclear gene expression mediated by redox state (Shaikhali et al., 2008; Shaikhali et al., 2012; Viola et al., 2013). However, there are two main questions about the role of ROS in MRS. The first one is related with the specificity of the response (i.e., how the information about the origin of the signal is retained and transmitted to the nucleus). The second one is related with the possibility that these molecules, once generated in mitochondria, travel through the cell to reach the nucleus and directly modify the activity of transcription factors. Recently, a model for H_2_O_2_ migration across the cell concludes that this type of signaling is unlikely to work in practice (Vestergaard et al., 2012). However, even if H_2_O_2_or other ROS could not be the signal *per se*, redox changes produced by these molecules in other compounds may act as signal relayers. Plant mitochondria contain many proteins with redox-active thiols that could sense H_2_O_2_ *in situ* and transduce ROS signaling. However, if this kind of redox regulation is involved in MRS is not fully understood. Another well characterized ROS is the superoxide anion (Zarepour et al., 2012). Superoxide is specifically reactive to iron-sulfur (Fe-S) clusters. In this way, aconitase is prone to suffer superoxidative inactivation (Morgan et al., 2008). Also, nitric oxide (NO) production was shown to inactivate aconitase and produce an increase in AOX1a expression (Polidoros et al., 2005; Gupta et al., 2012; Watanabe et al., 2010).

Other redox molecules that may be involved in MRS are glutathione and ascorbate. The last step of ascorbate synthesis is functionally and physically linked to the mitochondrial respiratory chain. Although ascorbate levels in plant cells are tightly controlled at the level of synthesis, recycling, degradation and transport (Green and Fry, 2005), chemical inhibition of respiration produces a strong decrease in total ascorbate levels (Millar et al., 2003). Perturbations in ascorbate metabolism lead to severe changes in gene expression, mostly related with plant-pathogen interactions, hormones and photosynthesis light reactions (Kiddle et al., 2003; Pastori et al., 2003; Kerchev et al., 2011). A role of ascorbate in MRS, though possible, has not been demonstrated. Interestingly, transcriptomic changes produced by mutations in ABI4, VTC2 and VTC4 proteins (the last two affect plant ascorbate levels) show a strong overlap suggesting the existence of a link between ascorbate and ABA signaling (Kerchev et al., 2011). Since ABI4 is involved in MRS, ascorbate levels may influence MRS acting on the ABI4 pathway. In the case of glutathione, no reports linking changes in its redox status with MRS are available. It should also be mentioned that, apart from redox molecules, changes in the levels of other metabolites, like carbon compounds or ATP, due to mitochondrial perturbations, may be used by the cell to adjust nuclear gene expression. The recently described effect of citrate in nuclear gene expression (Finkemeier et al., 2013) is an example of how changes in mitochondrial metabolism may be transduced to the nucleus through changes in metabolite levels.

How the specificity of the signal is transduced to the nucleus is difficult to envisage. In an extreme case, a molecule that is exclusively present in mitochondria must travel to the nucleus to induce MRS. In this sense, Møller and Sweetlove (2010) postulated that these specific molecules may be peptides derived from the degradation of mitochondrial proteins under stress conditions. Although appealing, evidence for the existence of this pathway is lacking. A different possibility is that there is not one specific signal, but rather a mitochondrial signature composed of many different unspecific signals and it is the integration of these signals in the nucleus that provides specificity to the response. This may have allowed the utilization and adaptation of preexisting signaling pathways to establish the communication between both compartments of the cell.

## CONCLUSION

**FIGURE 3:**
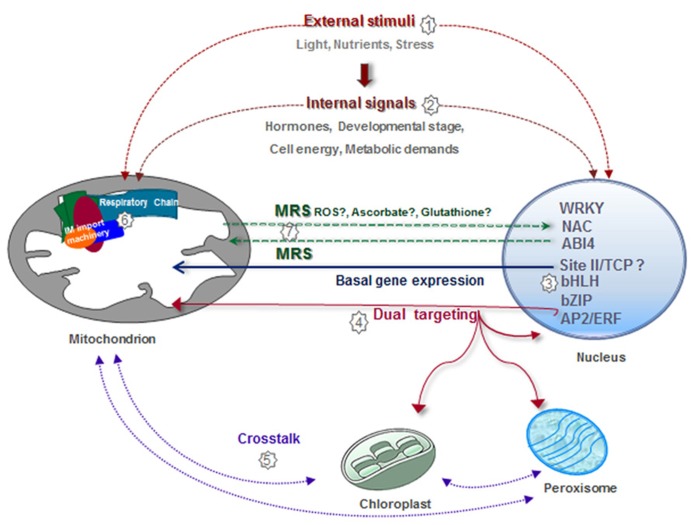
**Integrative view of different regulatory pathways involved in mitochondrial biogenesis.** Mitochondrial biogenesis is regulated by numerous external factors, such as nutrient availability, environmental stimuli, light/dark conditions, diurnal cycle, and oxidative stress situations, among others **(1)**. Mitochondrial biogenesis is also internally regulated by different stages of development, organ/tissue type, hormones, and cell energy and metabolic demands **(2)**. The expression of a majority of nuclear genes for mitochondrial proteins is controlled by elements known as site II that are recognized by TCP transcription factors **(3)**. Site II elements are either responsible for basal gene expression or modify the magnitude of the response under different growth conditions. Other transcription factors involved in the expression of nuclear genes encoding mitochondrial components belong to the bZip, AP2/ERF, and bHLH families **(3)**. Evidence exists demonstrating the presence of proteins dual targeted to different organelles, which may act to coordinate the activities of these organelles **(4,5)**. Physical and functional interactions between the inner membrane (IM) import machinery and complexes I, III, and IV help to adjust gene expression and protein assembly **(6)**. Mitochondria generate signals to modify nuclear gene expression according to organelle demands **(7)**.

Mitochondria are among the most plastic organelles of cells, alternating their number, architecture, and distribution throughout the cytosol in order to optimize their function according to specific energetic needs. In agreement with the central role of mitochondria as energy suppliers for cellular processes, mitochondrial biogenesis is a highly regulated process. In **Figure 3**, we summarize the different regulatory pathways which converge for the correct assembly of functional mitochondria (**Figure 3**). Many nuclear genes encoding mitochondrial components, and especially those encoding components of the respiratory chain, are coordinately transcribed due to the presence of common regulatory elements in their promoters. This reassures the synthesis of a basic set of mitochondrial components and produces global changes associated with cellular requirements for growth and defined metabolic states. More specific interactions between certain transcription factors and defined genes help to fine-tune this general response. Superimposed on this, external (e.g., stress) or internal (i.e., mitochondrial status) signals impact on the expression of specific sets of genes involved in the adaptation to the new situation. Many of the changes in nuclear gene expression take place at the transcriptional level. However, although examples of other forms of regulation are not abundant, it is likely that post-transcriptional mechanisms also have a role. Once imported, many nuclear-encoded proteins have to assemble with their counterparts synthesized in mitochondria to constitute functional complexes. The final amount of assembled complexes will depend on the availability of the respective subunits. It can be envisaged that limiting factors may be different under different conditions and this will provide a complex way of regulation by multiple inputs. Assembly may be also regulated by post-translational modification of proteins, but limited information is available on this. Finally, supercomplexes may be assembled or disassembled according to specific demands. The molecules and signals involved in this complex regulatory network are still largely unknown.

## Conflict of Interest Statement

The authors declare that the research was conducted in the absence of any commercial or financial relationships that could be construed as a potential conflict of interest.
